# Inguinal hernia recurrence: Classification and approach

**DOI:** 10.4103/0972-9941.27728

**Published:** 2006-09

**Authors:** Giampiero Campanelli, Diego Pettinari, Marta Cavalli, Ettore Contessini Avesani

**Affiliations:** Department of Surgical Sciences, University of Milano, Policlinico Hospital I.R.C.C.S, Pad. Beretta Est, Milano, Italy

**Keywords:** Classification, groin hernia, recurrence, treatment

## Abstract

The authors reviewed the records of 2,468 operations of groin hernia in 2,350 patients, including 277 recurrent hernias updated to January 2005. The data obtained - evaluating technique, results and complications - were used to propose a simple anatomo-clinical classification into three types which could be used to plan the surgical strategy:

- Type R1: first recurrence ‘high,’ oblique external, reducible hernia with small (<2 cm) defect in non-obese patients, after pure tissue or mesh repair
- Type R2: first recurrence ‘low,’ direct, reducible hernia with small (<2 cm) defect in non-obese patients, after pure tissue or mesh repair
- Type R3: all the other recurrences - including femoral recurrences; recurrent groin hernia with big defect (inguinal eventration); multirecurrent hernias; nonreducible, linked with a controlateral primitive or recurrent hernia; and situations compromised from aggravating factors (for example obesity) or anyway not easily included in R1 or R2, after pure tissue or mesh repair.

Inguinal hernia recurrence is still too frequent in the large published series.[1,2] Whatever the surgical technique used, its incidence is often inaccurately recorded because of inadequate follow-up in terms of methodology used, its duration or proportion of patients followed up as compared to the ‘operated-again’ patients. The rate of recurrence appears to be between 0.2 and 10%; these rates well explain the importance of the problem,[3] considering that hernia is a common problem around the world.

Recurrences occur as a result of different causes and promoting factors: old age, obesity, type of anesthesia, suture material used, way of dealing with the sac, type of repair and postoperative complications.[4] Today, especially in the era when use of prosthesis is common, our attention is as focussed on the anatomical, biological and mechanical factors as it is on the adequacy of repair, choice of technique used and the operative errors.[5,6]

The aim of this study is to propose a classification of recurrences of groin hernia that allows logical choice of repair to be utilized leading to a reduction in the re-recurrence rate.

## MATERIALS AND METHODS

From January 1992 until January 2005, 2,468 hernia repairs were performed in 2,350 patients in the Department of Surgical Sciences of Milan University at Policlinic Hospital, Pavilion Beretta Est. The 2,371 groin hernias (96.06% of the total hernia repairs undertaken) were distributed as 2,094 (88.3%) primary hernia repairs and 277 (11.7%) recurrent hernia repairs. Out of the recurrences, 90% occurred in male and 10% in female patients. The median age of the patients was 52 years and the median weight of the patient was 70 kg. There were 70% first-time recurrences, 25% second-time recurrences, 4% third-time recurrences and 1% fourth-time recurrences. For repair of the recurrences, we employed pre-peritoneal repair (Wantz modified) in 174 cases (62.81%), the Gilbert's plug technique by an anterior approach in 26 cases (9.38%) and Stoppa repair in 77 cases (27.79%). Local anesthesia was used in 101 patients (36.46%), 167 (60.28%) patients were operated under general anesthesia and 9 patients (3.24%) under spinal anesthesia.

Our follow-up ranged from 6 months to 12 years. We had an overall rate of postoperative complications of less than 3% (hematoma, edema, postoperative neuralgia, ecchymosis) and we had a recurrence rate of 4.4%.

In the last 9 years, the operations have been performed according to our classification, which allowed us to preoperatively determine the best choice for repair for an individual patient.

## DISCUSSION

The choice of an optimal strategy and surgical technique is probably more important in the treatment of recurrent hernias than in other areas of hernia surgery. The repairs in recurrent hernias have to be ‘personalized’ taking into account the technique/s that failed at earlier operation/s, the number of times the hernia has recurred, type of hernias, as well as general patient-related factors that affect the choice of anesthesia. In practice, we give importance to the preoperative examination regarding the size of hernia defect (less than 2 cm or more - small or big), site of the defect (‘high - external oblique’ near the reconstructed internal inguinal ring: where the previous repair has failed if it was a pure tissue repair, where the sac was not dealt with adequately or if the original repair was a mesh repair if the ‘mesh ring’ was too large; ‘low - direct’ above the pubis, where the previous repair failed because the sutures at this level were too many or too tight in pure tissue repair or if the distal part of the mesh was not adequately fixed or overlapped on the pubis in a mesh repair; ‘whole wall,’ where both after pure tissue repair and mesh repair, there is a complete failure of the previous operation), reducibility of the recurrent hernia, number of recurrences and patient's body habitus (obesity or overweight). The importance of a correct strategy (tactical) and technique seems to us even greater when the surgery for a recurrent hernia has to be planned as an outpatient procedure under local anesthesia. With reference to the site, Lichtenstein's consideration regarding the distribution is important:[7]

- 47% at pubic tubercle,
- 40% at internal ring,
- 13% entire wall.

We then tried to determine some parameters that allowed us to decide beforehand which patients were suitable for local anesthesia as outpatients and also the approach (anterior or posterior) and the surgical technique. With our experience, we propose an original anatomo-clinical classification of recurrences that can help the surgeon in individuating the choice of operation.

The recurrent hernias are divided into three types:

- Type R1: first recurrence ‘high,’ oblique external, reducible hernia with small (<2 cm) defect in non-obese patients, after pure tissue or mesh repair.
- Type R2: first recurrence ‘low,’ direct, reducible hernia with small (<2 cm) defect in non-obese patients, after pure tissue or mesh repair.
- Type R3: all the other recurrences - including femoral recurrences; recurrent groin hernia with big defect (inguinal eventration); multirecurrent hernias; non-reducible, linked with a controlateral primitive or recurrent hernia; and situations compromised from aggravating factors (for example obesity) or anyway not easily included in R1 or R2, after pure tissue or mesh repair.

This classification allows proposing the following decision-making choices:

- In cases with an R1 recurrence, we prefer a Gilbert's plug repair[3,5,6] by an anterior approach, under local anesthesia.
- In cases with an R2 recurrence, we perform a preperitoneal modified Wantz repair,[8–10] under local anesthesia. If R2 recurrence is after a previous preperitoneal mesh repair, we choose an anterior approach such as Lichtenstein, Gilbert or Trabucco. In both cases, only local anesthesia is used and the patient is discharged immediately.[8,11]
- In case of an R3 recurrence, we prefer a Stoppa's operation by preperitoneal approach, the Wantz technique for unilateral hernias or the laparoscopic technique.

This classification and the proposed approach take into consideration the fact that majority of the recurrences are after an anterior approach (pure tissue in past repairs and mostly mesh repair nowadays). It is very well known that a re-operation by the same route is likely to be difficult as a result of the presence of fibrotic tissue; the latter may make the dissection difficult or may cause damage to the cord structures.[4,8,12,13] Moreover, these conditions make surgery under local anesthesia difficult. For these reasons, we don't believe that all recurrences can be approached in this manner.

Nevertheless, in some cases such as the case R1, where most of the previous repair is still strong except for a small defect near the internal ring, the dissection can be easily performed only around the internal ring itself, the sac isolated and inverted and a plug inserted. This can be easy to perform, rapid and effective even in the case of previous mesh repair by an anterior approach.[14,15]

In cases with an R2 recurrence (with hernia's defect sited exactly at the superior edge of pubic tubercle), if a plug or mesh is to be inserted by an anterior approach, one has to undertake an extensive dissection of muscle-fascial plane and spermatic cord, with all possible negative consequences. Moreover, this defect is a ‘virtual space’ because it is in a site where normally there is no passage. This is different from the R1 type, where the internal ring is the natural passage of the cord; so the route is not virtual but real. For this reason, we prefer not to utilize an anterior approach with plug repair, ‘forcing’ the preperitoneal space and allowing a less-than-complete repair. For this reason, in R2 recurrences we prefer to perform a preperitoneal Wantz repair, which can be undertaken under local anesthesia. This avoids the risk of damage to cord structures and nerves as the surgery is performed in previously undisturbed planes[16,17] and also allows for a complete repair. We accept that for the preperitoneal repair to be undertaken under local anesthesia, the surgeon has to be well versed with the anatomy of this space and has to be experienced enough to keep the operative time short.[17]

In the presence of an R3-type recurrence, we always prefer general anesthesia and perform a preperitoneal repair. The laparoscopic repair by either a transperitoneal or a totally extraperitoneal route has been suggested for this type of recurrence. A laparoscopic repair allows the benefits of an early discharge, rapid resumption of activity and reduced postoperative pain. For surgeons who are not experienced in laparoscopic hernia repair, the most suitable repair for the R3 recurrences is a Stoppa or Wantz repair.

## CONCLUSIONS

In treating patients with recurrent hernias, we always choose to use prosthetic materials as their use has been supported by a large number of series. To allow reduction in the cost of surgery, we prefer to select a particular type of repair based on our anatomo-clinical classification and perform the surgery wherever possible under local anesthesia. This cuts down the period of hospitalization and allows rapid resumption of work by the patient.
